# Genome-wide Characterization of the JmjC Domain-Containing Histone Demethylase Gene Family Reveals GhJMJ24 and GhJMJ49 Involving in Somatic Embryogenesis Process in Cotton

**DOI:** 10.3389/fmolb.2022.888983

**Published:** 2022-04-27

**Authors:** Yan Li, Shouhong Zhu, Jinbo Yao, Shengtao Fang, Tengyu Li, Bei Li, Xinyu Wang, Mingyang Wang, Lanxin Wu, Jingwen Pan, Xuemei Feng, Wei Chen, Yongshan Zhang

**Affiliations:** ^1^ State Key Laboratory of Cotton Biology, Institute of Cotton Research, Chinese Academy of Agricultural Sciences, Anyang, China; ^2^ School of Agricultural Sciences, Zhengzhou University, Zhengzhou, China; ^3^ Shandong Denghai Shengfeng Seed Industry Co., Ltd., Jining, china

**Keywords:** epigenetics, histone demethylation, JmjC domain-containing genes, embryogenic callus, cotton

## Abstract

The Jumonji C (JmjC) domain-containing protein family, an important family of histone demethylase in plants, can directly reverse histone methylation and play important roles in various growth and development processes. In the present study, 51 *JmjC* genes (*GhJMJs*) were identified by genome-wide analysis in upland cotton (*Gossypium hirsutum*), which can be categorized into six distinct groups by phylogenetic analysis. Extensive syntenic relationship events were found between *G. hirsutum* and *Theobroma cacao*. We have further explored the putative molecular regulatory mechanisms of the *JmjC* gene family in cotton. *GhJMJ24* and *GhJMJ49* were both preferentially expressed in embryogenic callus compared to nonembryogenic callus in cotton tissue culture, which might be regulated by transcription factors and microRNAs to some extent. Further experiments indicated that GhJMJ24 and GhJMJ49 might interact with SUVH4, SUVH6, DDM1, CMT3, and CMT1 in the nucleus, potentially in association with demethylation of H3K9me2. Taken together, our results provide a foundation for future research on the biological functions of *GhJMJ* genes in cotton, especially in somatic embryogenesis in cotton tissue culture, which is crucial for the regeneration of transgenic plants.

## Introduction

Histone modifications, such as methylation, demethylation, phosphorylation, and ubiquitination, are important epigenetic modifications that play important roles in modulating gene transcriptional activity. Among various histone modifications, histone methylation has been reported to occur on Arg and Lys residues and is involved in multiple biological processes, such as target gene expression regulation, chromatin status modification, and epigenetic memory (Jenuwein and Allis, 2001; Martin and Zhang, 2005). Genome-wide analysis unraveled that about two-thirds of all the annotated *Arabidopsis* genes can be monomethylated, dimethylated, or trimethylated at histone H3 Lys residues (Zhang et al., 2009).

Histone methylation can be reversed by histone demethylases in organisms. Plants have two known families of histone demethylases, which play important roles in the demethylation of Lys residue. *Lysine Specific Demethylase1* (*LSD1*, also known as *KDM1* or *KDM1A*), a member of the flavin-dependent amine oxidase gene family, was the first identified histone demethylase to regulate gene activation and repression (Shi et al., 2004). Genes in the other class of histone demethylases constitute the Jumonji C (JmjC) domain-containing protein family, which directly reverse histone H3 lysine 4 (H3K4) and H3 lysine 9 (H3K9) modifications through oxidative reactions that are dependent on ferrous ions (Fe(II)) and α-ketoglutarate (αKG) (Elkins et al., 2003; Trewick et al., 2005). In the α-ketoglutarate binding sites, Thr/Phe/Ser and Lys were conserved and in the Fe(II) binding sites, two His and Glu/Asp were conserved (Huang et al., 2016). In addition to the JmjC domain, some other domains, such as JmjN, C5HC2 zinc-finger, FYRN and FYRC domains, were also found in some JmjC family members, which were shown to be involved in various cellular processes, such as catalytic activity and protein interactions (Mosammaparast and Shi, 2010; Qian et al., 2019).

Plant *JmjC* genes have been proven to play crucial roles in growth, development, and in response to environmental stresses (Klose et al., 2006; Kouzarides, 2007). To date, the functionalities of some plant *JmjC* gene members have been well investigated, mainly in model plants such as *Arabidopsis* and rice. In *Arabidopsis*, *AtJMJ11*/*ELF6* (*EARLY FLOWERING 6*) and *AtJMJ12*/*REF6* (*RELATIVE OF EARLY FLOWERING 6*), a pair of paralogs in *Arabidopsis*, played distinct functions in regulating flowering time (Noh et al., 2004; Yu et al., 2008). *AtJMJ13*, an H3K27me3 demethylase, may act as a temperature- and photoperiod-dependent genetic factor controlling plant flowering time by enhancing the expression of *FLOWERING LOCUS T* (*FT*) (Zheng et al., 2019). *AtJMJ14*, an active histone H3K4 demethylase, was found as a repressor for early flowering by suppressing the expression of *FT* and its homologs (Lu et al., 2010; Yang et al., 2010), acting in concert with two new NAC transcription factors, *NAC050* and *NAC052* (Ning et al., 2015). *AtJMJ15* was attributable to the alleviation of the salt stress symptoms relative to wild type control plants, while its loss-of-function mutant displayed increased sensitivity to salt treatment (Shen et al., 2014). Corroborating observation was also made in AtJMJ17, another histone H3K4 demethylase in *Arabidopsis*, which played a positive role in response to dehydration stress (Huang et al., 2019). AtIBM1/JMJ25 was a histone H3K9 demethylase, which was found to play an essential role in gene activation by preventing the spreading of gene silencing from heterochromatin. Moreover, mutations in *jmj25* induced a variety of developmental phenotypes, such as leaf deformation, abnormal flowers, pollen defects, and reduced fertility (Saze et al., 2008). In addition, AtJMJ29 was involved in trichome development by directly targeting GL3 and removing H3K9me2 on the GL3 locus. In rice, a loss-of-function mutant in *OsJMJ706* enhanced dimethylations and trimethylations of lysine 9 of histone H3 (H3K9) *in vitro* that impacted on the number of floral organs per spikelet in rice (Sun and Zhou, 2008). *OsJMJ705*, an H3K27me3 demethylase, regulates shoot development in rice by interacting with *WUSCHEL-RELATED HOMEOBOX11* (*WOX11*) (Cheng et al., 2018). *OsJMJ703*, was demonstrated to affect rice panicle morphology when overexpressed while its knock-down mutant displayed an earlier flowering phenotype (Song et al., 2018).

Cotton is the most important fiber crop with significant economic value. For the improvements in cotton productivity and fiber quality, transgenic cottons have been developed as the first commercial transgenic crops, by leveraging *Agrobacterium*-mediated genetic transformation and plant regeneration via somatic embryogenesis (Zhang, 2019). Despite the overt success in commercial exploitation, broad application of transgenic technology in cotton is constrained by the genotypic variation in the capability of plant regeneration from tissue culture as the vast majority of elite cotton germplasms are recalcitrant to the present transgenic protocols (Leelavathi et al., 2004). The somatic embryogenesis process that is a key step in the regeneration of transgenic plants is now known to be regulated by manifold epigenetic mechanisms, especially DNA methylation and histone modification (De-la-Pena et al., 2015; Ikeuchi et al., 2016). The callus derived from tissue culture following the inoculation with *Agrobacterium* harboring the target gene construct can be classified into non-embryogenic callus (NEC) and embryogenic callus (EC), with the possibility that the former may be convert into the latter leading to somatic embryo development. A recent study showed that the transformation from NEC to EC was associated with the RNA-dependent DNA methylation (RdDM) and the H3K9me2-dependent pathway. Inhibiting DNA methylation using zebularine treatment in NEC increased the number of somatic embryos in cotton transformation (Li et al., 2019). Considering the well-known roles of JmjC in modulating histone methylation in plants, we set out to investigate the potential involvement of cotton JmjC gene family in somatic embryogenesis during cotton tissue culture. In this study, we identified 51 putative *JmjC* genes in *G. hirsutum* genome, herein termed as *GhJMJ*, and systematically analyzed their gene structure, phylogenetic relationships, conserved motifs, syntenic relationship and spatial expression profiles. The comparative analysis of the expression levels of the GhJMJ genes in EC and NEC were further determined by RT-PCR. Transcription factor binding sites (TFBS) in the putative promoters and microRNA (miRNA) target sites of *GhJMJs* were also predicted. Subcellular localization assays and interaction network analysis unraveled that two key GhJMJs including GhJMJ24 and GhJMJ49 were present in the nucleus where they function by interacting with SUVH4, SUVH6, DDM1, CMT3, and CMT1. This study may shed more light on the functionality of the *JmjC* gene family in cotton, with respect to plant regeneration via somatic embryogenesis in cotton tissue culture and gene transformation.

## Materials and Methods

### Identification and Sequence Analysis of *GhJMJ* Gene Members

By using TBLASTP and the protein sequences of 21 *Arabidopsis* and 20 rice JmjCs derived from the Arabidopsis Information Resource (http://www.arabidopsis.org/) and the Rice Genome Annotation Project (http://rice.plantbiology.msu.edu/index.shtml), respectively, as query sequences, cotton homologs were searched against the genome sequences of *G. hirsutum* (JGI,v1.1), *G. raimondii* (JGI, V2.0), and *G. arboreum* (CRI, v1.0) which were downloaded from CottonFGD (http://www.cottonfgd.com) (Yu et al., 2021; Zhu et al., 2017). For comparison, the genome database of cacao (*Theobroma cacao* v1.1) obtained from JGI (https://phytozome.jgi.doe.gov/pz/portal.html) was also searched. All the sequences with an e-value below 10^−10^ were regarded as candidate proteins. The resulting protein sequences were then verified for the presence of the JmjC domains, including PF02373, SM00558, PTHR12549 by using Pfam (El-Gebali et al., 2019) (http://pfam.sanger.ac.uk/search), SMART (Letunic et al., 2015) (http://smart.embl-heidelberg.de/), and PANTHER (Mi et al., 2017) (http://www.pantherdb.org/tools/sequenceSearch.do). In the case of a potential gene with multiple alternative splice variants, the longest one was chosen for the candidate protein. The molecular masses and isoelectric points were calculated based on the Compute pI/Mw tool of ExPaSy (Gasteiger et al., 2003) (https://web.expasy.org/compute_pi/). The subcellular localization of *GhJMJ* genes was predicted by using WoLF PSORT (http://wolfpsort.seq.cbrc.jp/).

### Phylogenetic Tree, Gene Structure, and Conserved Motif Analysis

All the DNA sequences were aligned by ClustalX 2.0, which was used for phylogenetic analyses in MEGAX by using the maximum likelihood (ML) method (Kumar et al., 2018). The Pfams of GhJMJ proteins were searched by Pfam, SMART, and PANTHER, and displayed by TBtools (Chen et al., 2020). The structures of the *GhJMJ* genes were acquired from the *G. hirsutum* genome annotation gff3 file by TBtools. The top 20 conserved motifs in the full-length amino acid sequences were identified by using MEME analysis (Bailey et al., 2006) (http://meme.nbcr.net/meme/), and the conservation level of motifs and the JmjC domain at each residue position were estimated by using the WebLogo application (http://weblogo.threeplusone.com).

### Chromosomal Location and Synteny Analysis

The chromosomal location of *GhJMJ* was investigated by using the position information from the *G. hirsutum* genome annotation gff3 file (JGI, v1.1). Based on the BLASTP results, MCScanX was used to find the duplicate type of *GhJMJ* and surveyed the intragenomic and intergenomic syntenic blocks of *G. hirsutum* with cacao or rice genomes (Wang et al., 2012). The synteny maps of the collinearity pairs within the *GhJMJ* gene family were constructed and illustrated by the Circos and TBtools programs (Krzywinski et al., 2009; Chen et al., 2020).

### Expression Analysis Based on RNA-Sequencing Data

Expression of *GhJMJ* genes was profiled based on the RNA-seq transcriptome data sets downloaded from CottonFGD corresponding to expression patterns in different tissues/organs in *G. hirsutum*. Gene expression levels were calculated according to FPKM (expected number of fragments per kilobase of transcript sequence per million of base pairs sequenced) values and the average FPKM value of each repetition was converted to log2 value, based on which Heatmaps were drawn by using HemI software (version 1.0) (Deng et al., 2014). Transcriptomic profiles of NEC and EC callus were obtained from the NCBI BioProject (ID PRJNA629328) as reported by a previous study (Wen et al., 2020). Gene expression log fold-change (logFC) values of *GhJMJs* and predicted interactive genes in EC were analyzed.

### Plant Materials and qRT-PCR

A highly regenerable *G. hirsutum* cultivar known as “Y668” was used for gene expression analysis. Samples were collected from NEC callus and EC callus at various developmental phases, and were immediately frozen in liquid nitrogen, and stored at −70°C until use. Each sample was prepared with three biological replicates. Total RNA was extracted from the samples by using the EASYspin Plus Plant RNA Kit (Aidlab, Beijing, China), following manufacturer’s instructions. First-strand cDNA synthesis was conducted by using PrimeScript RT reagent Kit with gDNA Eraser (Perfect Real Time) Cat# RR047A (Takara, Tokyo, Japan) and 1.0 µg RNA as template. This was followed by quantitative real-time PCR (qRT-PCR) that was conducted with TB Green Premix Ex Taq (Tli RNaseH Plus) Cat# RR420A (Takara) and the CFX96 Real-time PCR Detection system (Bio-Rad, Foster City, CA, United States). The primers used are listed in Supplementary Table S1. Relative expression levels were calculated by using the comparative threshold cycle (2^−ΔΔT^) method (Yin et al., 2018). Three technical replications were performed for each sample. Student’s t-test was used to analyze the significance between groups by GraphPad Prism 8.0 software.

### TFBSs Prediction

The 2,000 bp upstream sequences of all the identified *GhJMJ* genes were extracted by TBtools, from which TFBSs prediction was performed by using the Transcription Factor Prediction tool (http://planttfdb.gao-lab.org/), with the threshold *p*-value≤1 × 10^–6^. The annotation of all the predicted TFs was conducted by using GO enrichment analysis.

### MiRNA Target Sites Analysis

The microRNA (miRNA) sequences of cotton were obtained from the Plant MicroRNA database (http://bioinformatics.cau.edu. cn/PMRD/), and relevant publications (Yin et al., 2017). The miRNA binding sites in the coding regions (CDS) of GhJMJ genes were predicted by the psRNATarget server with default parameters, except maximum expectation (E) = 5.0 (http://plantgrn.noble.org/psRNATarget/home).

### Subcellular Localization

The full-length CDS without stop codon was fused to the N-terminus of green fluorescent protein (*GFP*) gene in the transient expression plasmid PAN580 to produce the 35S::GhJMJ-GFP construct. One other construct 35S::OsGhd7-RFP with red fluorescent protein (RFP) being fused to the nuclear marker OsGhd7 (Xue et al., 2008) was co-transformed into the isolated protoplasts of *A. thaliana* by the PEG4000-mediated method as previously described (Abel and Theologis, 1994). The 35S-GFP empty vector was used as a control. Fluorescence signals in protoplasts were detected by using a confocal laser scanning microscope Zeiss LSM 510 META (Zeiss, Jena, Germany) following a period of incubation for 15–20 h in the dark at room temperature.

### Protein Interaction (PPI) Network Prediction

In order to explore the interaction network of GhJMJ24 and GhJMJ24 protein, the homologous genes of *GhJMJs* in *G. raimondii* were used to construct a protein-protein interaction (PPI) network by using STRING (version 11.0) (Szklarczyk et al., 2019) (http://string-db.org/).

## Results

### Identification and Characterization of the *JmjC* Family Genes in Cotton

The protein sequences of the 21 Arabidopsis and 20 rice JmjC genes obtained from TAIR and RGAP (Supplementary Table S2), were served as queries for searching their homologs in Gossypium databases. As a result, a total of 51, 25, and 28 JmjC genes were identified in G. hirsutum, G. raimondii and G. arboretum, respectively (Supplementary Table S3). The JmjC gene family in these Gossypium species is considerably larger than those identified in rice and Arabidopsis (Lu et al., 2008). For convenience, each JmjC gene was named according to its chromosomal location. The primary molecular features of GhJMJ, such as the lengths of nucleotides and amino acids, the molecular weight (MW), isoelectric point (pI) and the subcellular location of the deduced proteins, are presented in Supplementary Table S3.

### Phylogenetic Analysis of *JmjC* Genes

**FIGURE 1:**
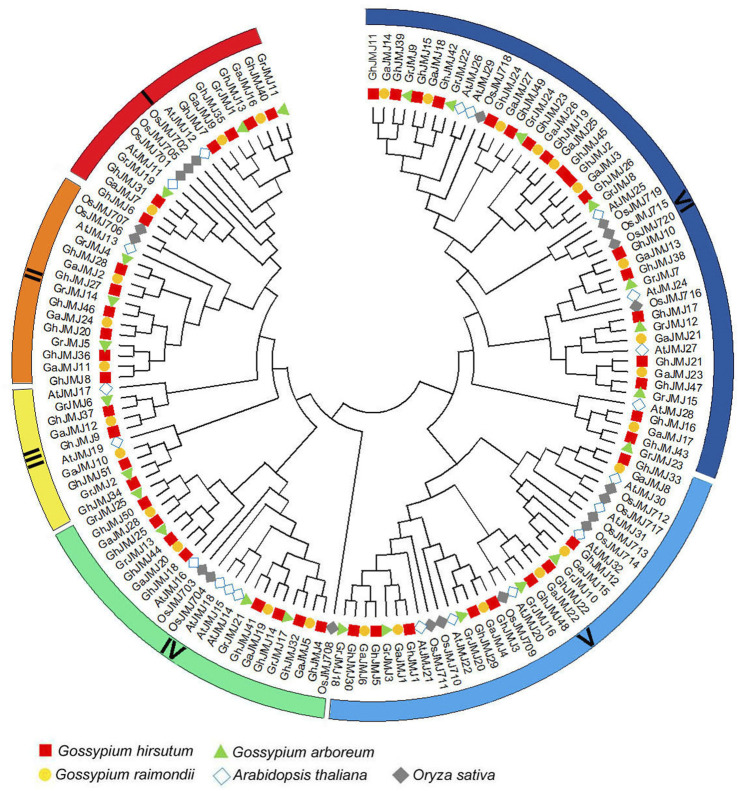
Phylogenetic relationships of JmjC family proteins in *Gossypium raimondii* (Gr)*, G. arboretum* (Ga)*, G. hirsutum* (Gh)*, Arabidopsis thaliana* (At) and *Oryza sativa* (Os). The colored arcs indicate different groups of JmjC proteins, and the combination of shape and color indicates different plant species.

To date, studies on *JmjC* genes have been focused on their biological functions in *O. sativa* and *A*. thaliana. To examine the evolutionary relationships of *JmjC* genes in cotton, *O. sativa* and *A. thaliana*, we constructed a phylogenetic tree of JmjC based on 145 full-length JmjC protein sequences, including 51, 25, 28, 20, and 21 sequences from *G. hirsutum*, *G. raimondii*, *G. arboreum, O. sativa*, and *A. thaliana*, respectively. It appears that *JmjC* could be categorized into six distinct groups rather than the five groups reported in recent studies (Zhang et al., 2020; Sun et al., 2021), which are denoted group I to group VI (Figure 1). Group VI, relative to other five groups, contains more genes from each of the five species. Group III contains the least genes and lacks one from *O. sativa*. As expected, the Gossypium genes have closer relationships with *Arabidopsis* than with rice. In addition, two members (*GhJMJ1* and *GhJMJ12*) of group V and *GhJMJ17* of group VI clustered together with the homologous genes in *G. arboretum* and *G. raimondii*, respectively. *GhJMJ33* in group V and two members (*GhJMJ19* and *GhJMJ23*) of group VI clustered together only with the homologs in *G. arboretum*. Except for *GhJMJ45* in group VI, which does not have orthologs in other *Gossypium* species, the *JmjC* genes in *G. hirsutum*, *G. raimondii*, and *G. arboreum* showed apparent homologous relationship in other four groups, consistent with their common evolutionary origin and the premise that the latter two species are closely related to the progenitor species of the former. These findings suggest that the *JmjC* genes in group V and group VI might have gone through some significant gene duplication and gene loss during the evolutionary process in cotton, and gene duplication may have afforded the opportunity for the duplicated genes diverge in function in cotton.

### Gene Structure, Conserved Domains, and Motifs of the *GhJMJ* Genes

**FIGURE 2:**
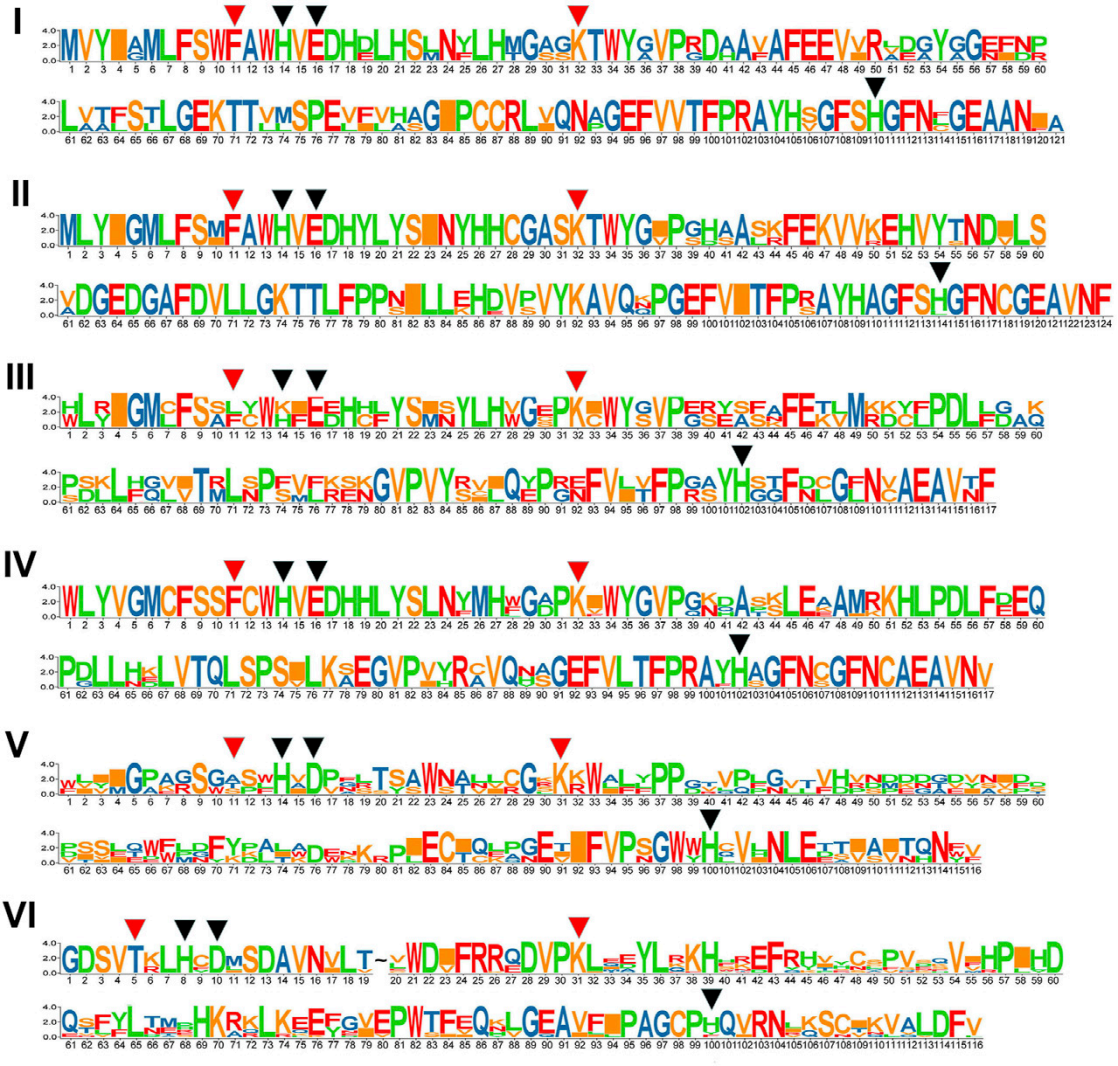
The conservation of JmjC domains in six *GhJMJ* gene subfamilies. Fe(II) binding sites are shown with red arrowheads, and αKG binding sites are shown with black arrowheads.

We compared the numbers, lengths, and arrangement of the exons and introns in the *GhJMJ* gene sequences (Supplementary Figure S1) to gain insight into their structural diversity. As shown in Supplementary Figure S1A, most genes contain numerous exons, except for *GhJMJ3* and *GhJMJ29,* which contain only two exons. Most of *GhJMJs* have upstream and downstream sequences, while *GhJMJ30* and *GhJMJ42* have no upstream or downstream sequences. There are eleven domains in GhJMJ including JmjC, JmjN, zf-C5HC2, FYRN, FYRC, AT_hook, zf-4CXXC_R1, WRC, PHD, F-box, and PLU-1 (Supplementary Figure S1B). JmjC was found in all GhJMJ proteins, and the αKG and Fe(II) binding sites in the JmjC domain showed high level of conservation (Supplementary Figure S2). Specifically, as shown in Figure 2, His (H), Glu (E), and His (H) were conserved in the Fe(II) binding sites, and Phe (F) and Lys (K) were conserved in the αKG binding sites in groups I, II, III, and V. In group VI, Phe (F) in the first αKG binding site was substituted with T (Thr), and Glu (E) in the Fe(II) site was substituted with D (Asp). However, in group V, Phe (F) residue of the first αKG binding site was substituted with Ala (A), or Thr (T) or Ser (S) (Figure 2). The JmjC domain is highly conserved within group, but diverge considerably among different groups. Moreover, cofactor binding sites of the JmjC domain have been modified in some group.

In addition, a total number of 20 distinct motifs were identified and designated as motif 1 to motif 20 in the GhJMJ gene family. The details of the motif sequences are shown in Supplementary Table S4. As shown in Supplementary Figure S3, motif 2 was identified in all GhJMJ proteins, but was less conserved than other motifs. Some motifs were specific to several subfamilies. For example, motifs 8 and 20 were found in all but the V and VI subfamilies. Interestingly, motifs 17, 19, 18, 10, 12, 7, 4, 1, and 9 were distributed in all members of group VI. Members in the same subfamily have similar domains and motifs, implying their possible conservation in functionality.

### Chromosomal Location and Synteny Analysis

**FIGURE 3:**
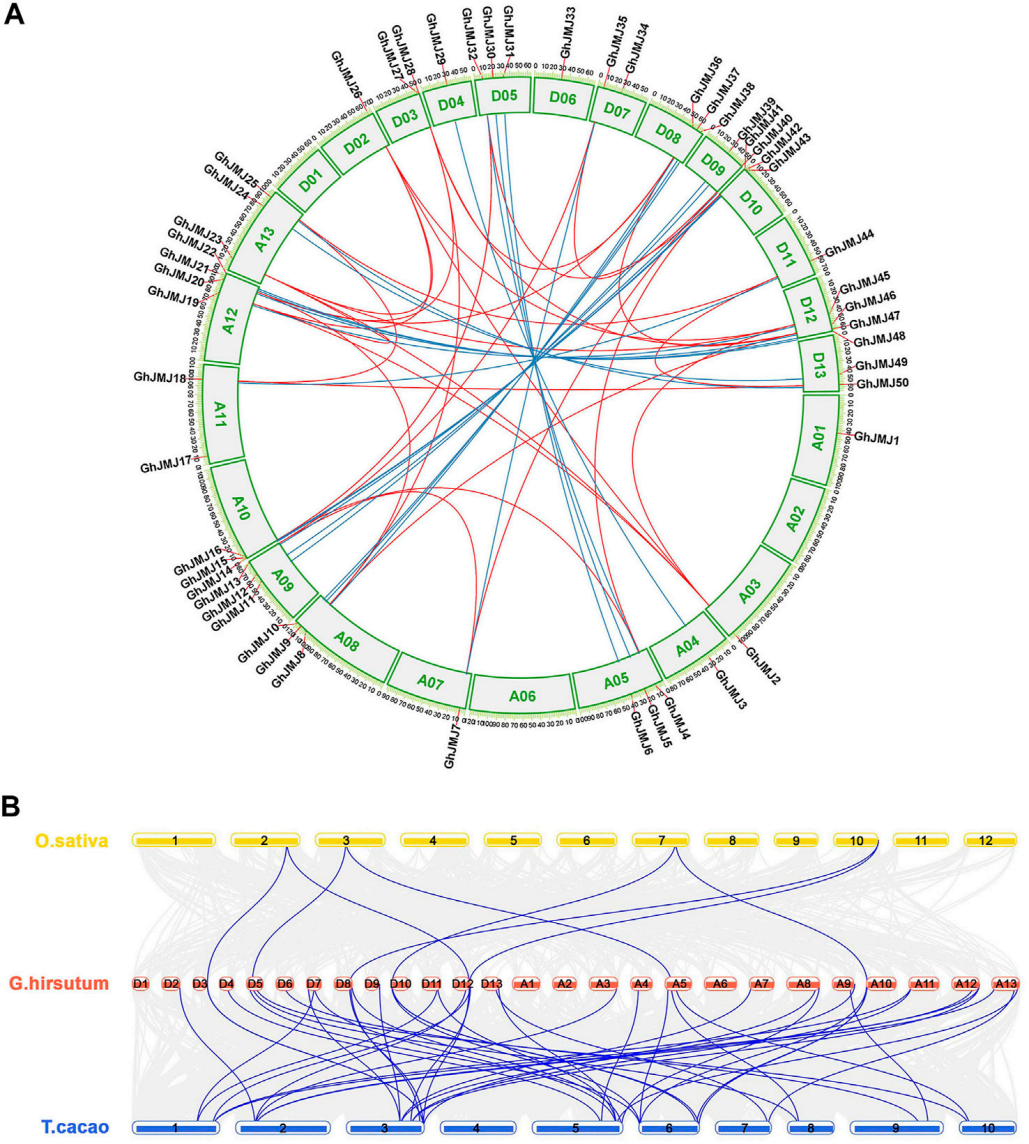
Collinearity analyses of *GhJMJ* genes in *Gossypium hirsutum* and synteny analysis of *JmjC* genes among *G. hirsutum*, *Oryza sativa*, and *Theobroma cacao*. **(A)**. The chromosomal distribution and collinearity relationships of *GhJMJ* genes. The blue lines link the orthologous genes, and red lines link paralogous pairs. **(B)**. Synteny analysis of *JmjC* genes between *G. hirsutum* and *O. sativa*, and between *G. hirsutum* and *T. cacao*.

**FIGURE 4:**
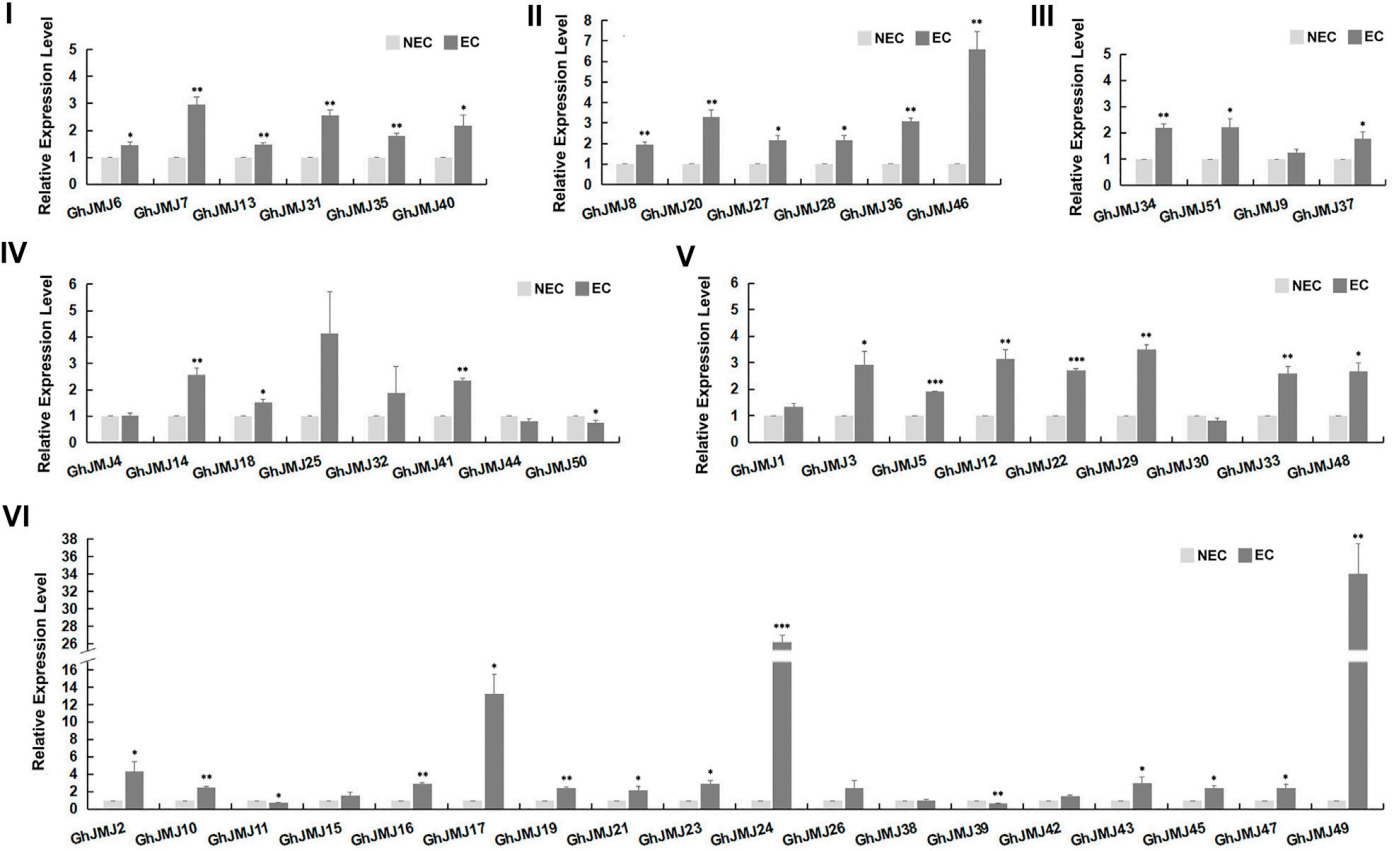
Expression patterns of six subfamilies of *GhJMJ* genes between NEC and EC tissues based on qRT-PCR. Three independent experiments were performed; the error bars indicated the SDs. *, **, and *** indicate significant differences compared with the control (NEC) at *p* < 0.05, *p* < 0.01 and *p* < 0.005, respectively, according to the Student’s t-test.

All the *GhJMJ* genes but one (*GhJMJ51*) were physically assigned to *G. hirsutum* chromosomes. The distribution of *GhJMJ* genes among cotton chromosomes appeared to be random (Figure 3A). Many members of the *GhJMJ* genes were duplicated in collinearity regions, and 20 orthologous gene pairs were identified between the At and Dt subgenomes (Supplementary Table S5). In particular, chromosome A12 and D12 each contained four *GhJMJ* genes and an abundant collinearity replication relationship existed with other chromosomes (Figure 3A). Gene duplication is commonly considered as the main driver of evolution, in the forms of dispersed, tandem, whole-genome, and segmental duplications. There were six dispersed genes (*GhJMJ1*, *GhJMJ9*, *GhJMJ17*, *GhJMJ28*, *GhJMJ34*, and *GhJMJ51*) in *G. hirsutum*, which might have arisen from transposition. The rest of the *GhJMJ* genes were predicted to be derived from whole-genome or segmental duplications that could be the dominant form of *GhJMJ* gene duplication in cotton (Figure 4A, Supplementary Table S6).

To explore the evolutionary relationship of *GhJMJ* genes beyond the *Gossypium* species, a collinear relationship of *G. hirsutum* with another dicotyledonous plant *T. cacao* and a monocotyledonous plant *O. sativa* by MCScanX (Figure 3B). Both specific loss and expansion of *GhJMJ* genes were found in these species. A total of 41 *GhJMJ* genes showed a syntenic relationship with those in *T. cacao*, but only seven *GhJMJ* genes showed a syntenic relationship with those in *O. sativa* (Figure 3B, Supplementary Table S7). Six *GhJMJ* genes, including *GhJMJ5*, *GhJMJ16*, *GhJMJ28*, *GhJMJ36*, *GhJMJ43*, and *GhJMJ46*, had syntenic relationships with both *T. cacao* and *O. sativa*, indicating that these genes are highly conserved during evolution.

### *GhJMJs* Are Differentially Expressed in EC and NEC Tissues

The expression of *GhJMJ* family members in different tissues/organs was examined by using previously published RNA-sequencing data and summarized in a heat map, including the root, stem, leaf, petal, calycle, pistil, torus, stamen, ovules, and fibers (Supplementary Figure S4, Supplementary Table S8). Further validation on gene expression at the NEC and EC stages was carried out by qRT-PCR (Figure 4). In groups I, II, and III, all expression levels were up-regulated in EC tissue. In group IV, four of the seven *GhJMJ* genes were up-regulated in EC tissue. All the *GhJMJ* genes but *GhJMJ30* in group V were up-regulated in EC tissue. Except for *GhJMJ11*, *GhJMJ38*, and *GhJMJ39*, all other *GhJMJ* genes in subfamily VI were up-regulated, among which a pair of homologs, *GhJMJ24* and *GhJMJ49* displayed higher relative expressions in EC tissue compared with NEC tissue. The transcriptomic profiles of *GhJMJs* in NEC and EC tissue were retrieved from a previous study (Wen et al., 2020). Fold changes in gene expression ranged from −1 to 3 in EC when compared to NEC (Supplementary Figure S4, Supplementary Table S13). It was shown that the expressions of *GhJMJ24* and *GhJMJ49* were significant up-regulated in EC (logFC >2), which was consistent with the qRT-PCR result discussed above. Taken together, it is conceivable to suggest that *GhJMJ24* and *GhJMJ49* in subfamily VI, might play important roles in the process of somatic embryogenesis by virtue of their high levels of expression in EC tissue.

### TFBS Predicted in the Promoter Regions of *GhJMJs*

**FIGURE 5:**
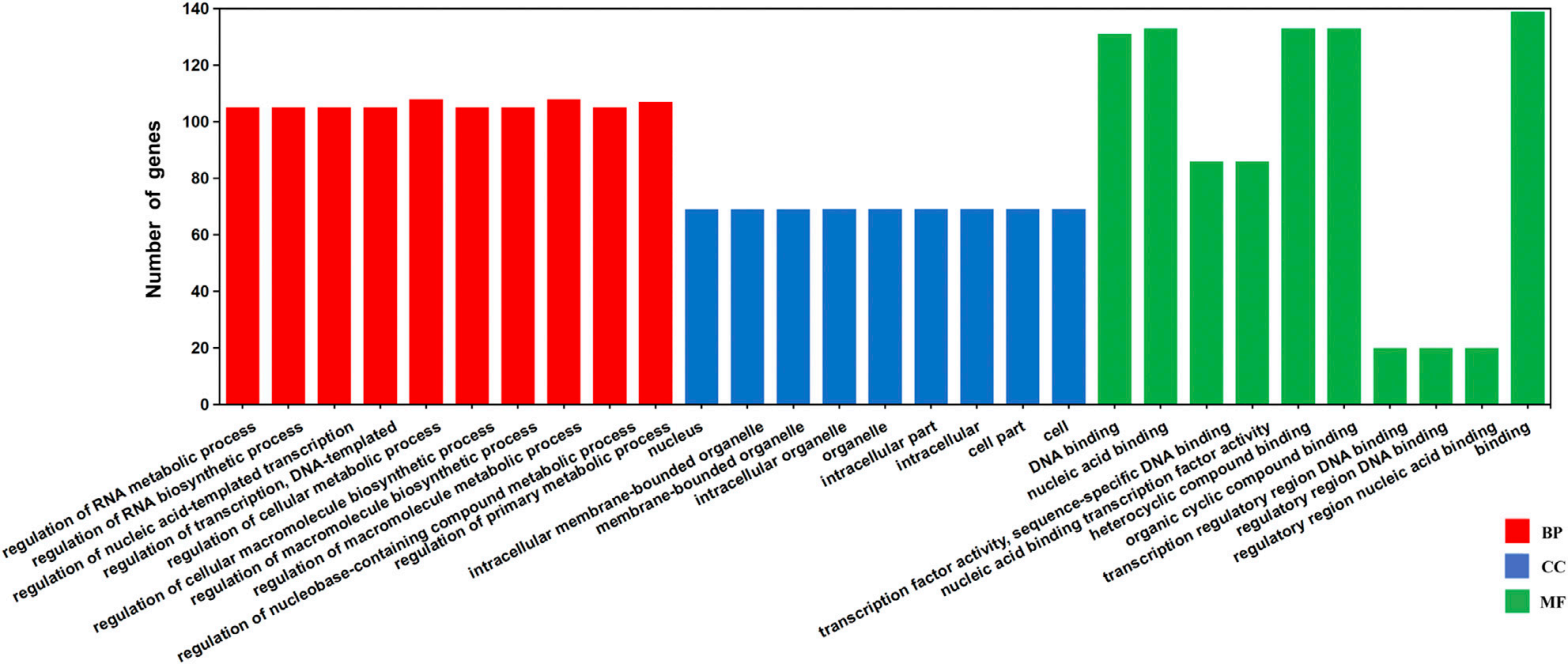
Gene Ontology (GO) annotation of the TFs binding to the putative upstream sequences of GhJMJs based on their cellular component, molecular function, and biological process. The Y-axis represents the number of genes in a sub-category. BP: Biological Process, CC: Cellular Component, MF: Molecular Function.

The potential regulatory TFs and their corresponding binding sites in *GhJMJs* were investigated. A total of 150 TFs belonging to 26 families and 545 putative TFBSs in the upstream sequences of 48 *GhJMJs* (except for GhJMJ4, GhJMJ17, and GhJMJ26) were identified (Supplementary Table S9). The DNA sequences of the putative TFBSs and the *p*-value candidate TFBSs were also given in Supplementary Table S9. Among the putative TFBSs, some were common in most organisms, such as the MYB, C2H2, and MADS families, while others such as NAC, Dof, BBR-BPC and LBD, were exclusively found in higher plants. GO annotation of 148 putative TFs potentially regulating *GhJMJs* was conducted which were enriched in 279 GO terms (Supplementary Table S10). The top scoring ten GO terms in each group were presented (Figure 5). It is apparent that most TFs were enriched in biological processes, such as acid-templated transcription and RNA biosynthetic/metabolic. In molecular function, TFs were mainly involved in DNA/nucleic acid binding, heterocyclic/organic cyclic compound binding. Interestingly, there was fewer TFs with involvements in cellular components.

**FIGURE 6:**
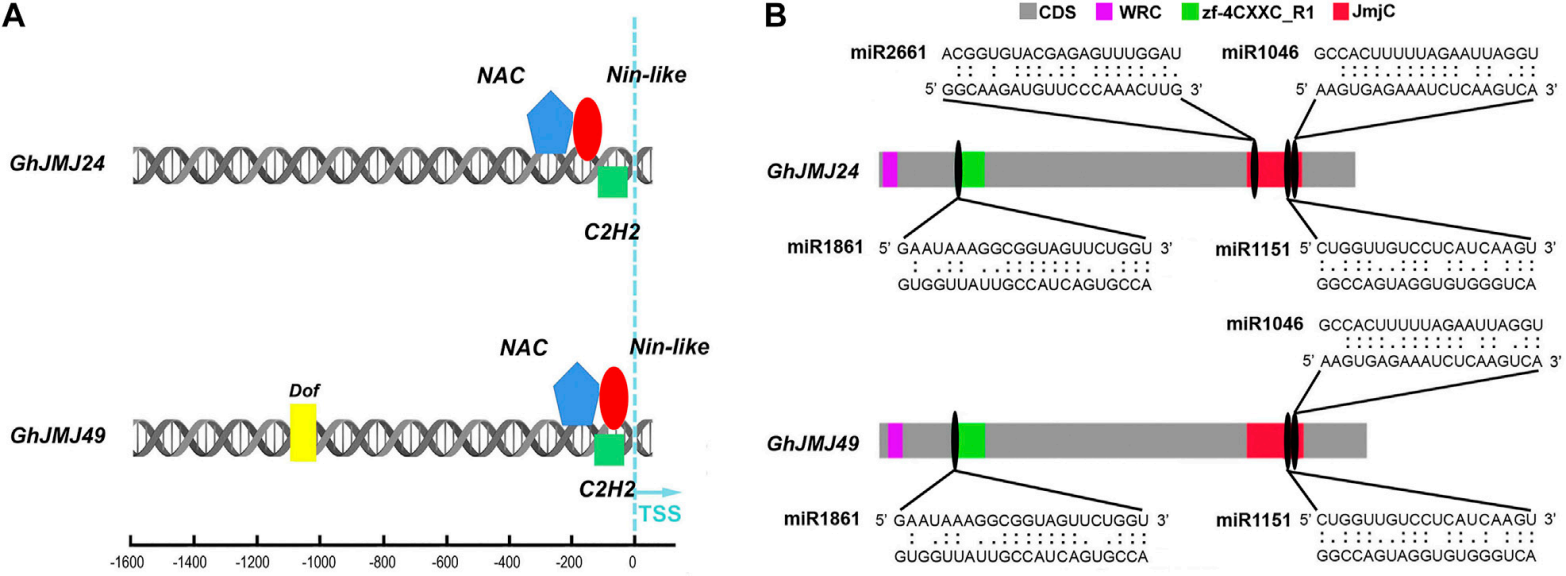
Schematic diagram of the transcription factor binding sites (TFBS) and miRNA complementary binding sites of *GhJMJ24* and *GhJMJ49*. **(A)**. TFBSs in the upstream sequences of *GhJMJ24* and *GhJMJ49*. Colored shapes depict putative TFBS. **(B)**. The miRNA-mRNA regulatory relationships in the domains of *GhJMJ24* and *GhJMJ49*. Grey boxes represent the CDS (open reading frames) of genes. JmjC domain, zf-4CXXC_R1 domain and WRC domain are shown in red, green, and purple boxes, respectively. The miRNA complementary binding sites are marked by black ovals, and complementary sequences (5–3′) are shown in the expanded regions.

Schematic diagrams representing the promoter regions of *GhJMJ24* and *GhJMJ49* showing putative TFBSs are presented in Figure 6A. There were three TFBS at *GhJMJ24* and *GhJMJ49* promoters close to the Transcription Start Sites (TSS), including NAC, C2H2 and Nin-like. In addition, Dof was predicted to bind at *GhJMJ49* promoter distal to TSS. Overall, these findings suggest that the expression of *GhJMJ* genes under the regulation of specific TFs could be attributable for cotton growth and development, especially in somatic embryogenesis in tissue culture.

### MiRNA Target Sites Analysis of *GhJMJs*

To investigate the miRNA-mediated post-transcriptional regulatory mechanisms of GhJMJs, potential miRNA target sites in CDS were searched by using the psRNATarget server. The presence of potential miRNA target sites was putatively found in all the *GhJMJs* (Supplementary Table S11). For example, ghr-miR414 was found to target GhJMJ1, GhJMJ12, GhJMJ16, and GhJMJ43, while ghr-miR156 may target the GhJMJ34 and GhJMJ51. MiR1045 may target the non-conserved domains of GhJMJ26 only. Multiple miRNA target sites were predicted in some *GhJMJ* genes; for instance, 13 and 12 miRNA target sites were respectively predicted in *GhJMJ24* and *GhJMJ49*, and the potential miRNA target sites in their conserved domains were shown in Figure 6B. Both *GhJMJ24* and *GhJMJ49* were targeted by miR1151, and miR1046 in the JmjC domain, and targeted by miR2661 in the zf-4CXXC_R1 domain*.* In addition, *GhJMJ24* was targeted by miR2661 in the JmjC domain.Taken together, these results infers that miRNA-mediated post-transcriptional regulation of *GhJMJs* might be involved in a variety of biological functions, which is intriguing and warrants further investigation.

### GhJMJ24 and GhJMJ49 Are Nucleus-Localized Proteins

**FIGURE 7:**
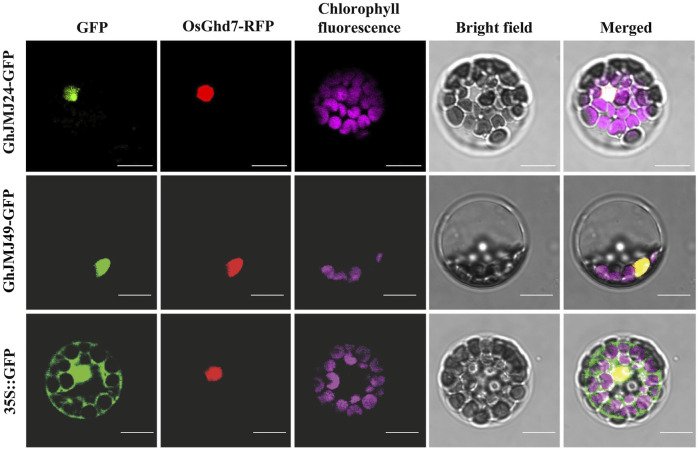
Subcellular localization of GhJMJ24 and GhJMJ49 in *Arabidopsis* protoplasts. Recombinant plasmid 35S::GhJMJ24-GFP, 35S::GhJMJ49-GFP and the empty vector 35S::GFP were transfected separately into *Arabidopsis* protoplasts with nuclear marker 35S::OsGhd7-RFP. Fluorescence signals from GFP, RFP, chlorophyll autofluorescence (pseudo-colored purple) and the merged images are shown. Scale bars, 10 μm.

The subcellular localizations of proteins are often closely associated with their functions. To facilitate the investigations into the subcellular localizations of GhJMJ24 and GhJMJ49, their GFP fusion constructs were co-expressed with the nuclear marker OsGhd7-RFP in *Arabidopsis* mesophyll protoplasts. As shown in Figure 7, GFP fluorescence are well overlapped well with RFP signals in both genes, revealing their nuclear localization feature. Such results are clearly congruent with the theoretical predictions (Supplementary Table S3) for their location in nucleus where they may play functional roles during cotton tissue culture and plant regeneration.

### Protein Interaction Network of GhJMJ24/GhJMJ49 and Gene Expression

**FIGURE 8:**
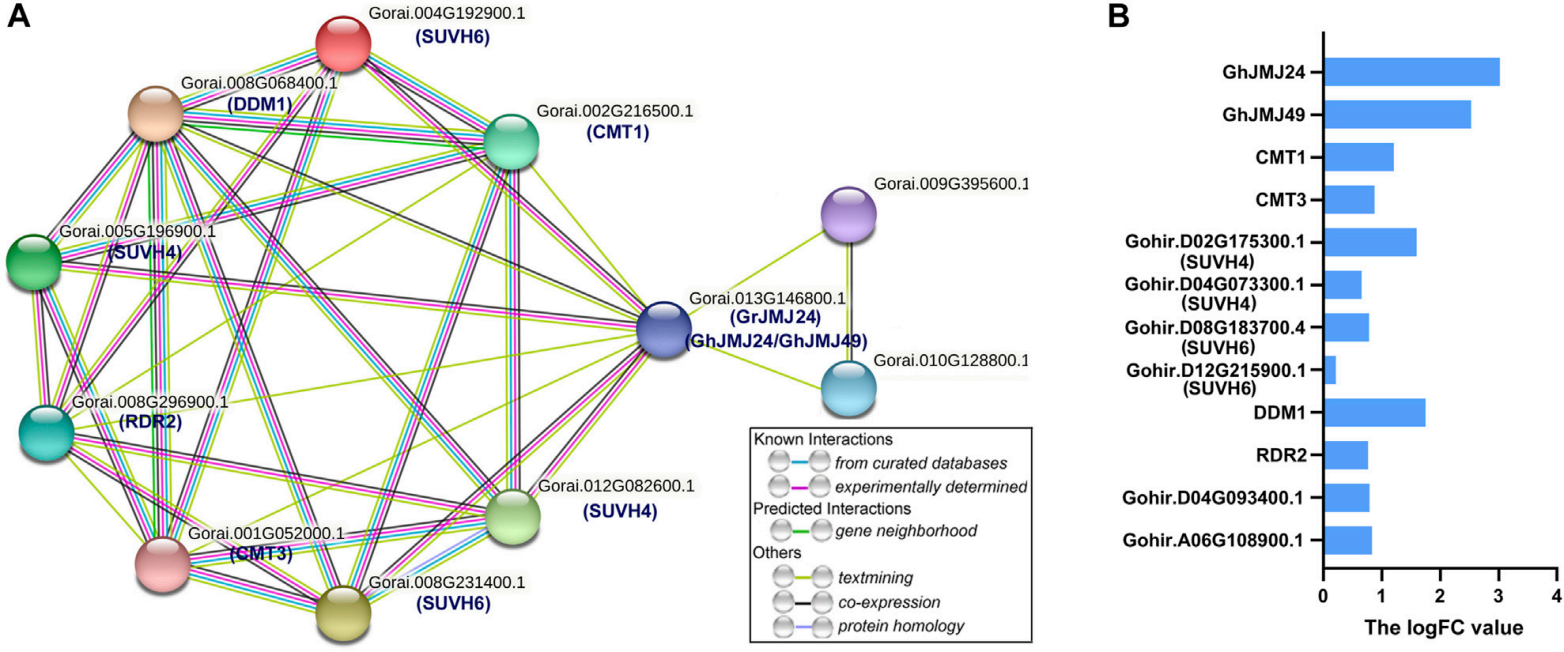
The protein interaction network of *GhJMJ24* and *GhJMJ49* and ten interactive genes expression. **(A)** The interaction network of GhJMJs homologues in *G. raimondii*. Network nodes represent proteins. Edges represent protein-protein associations and color shows association types. Known interactions: from curated databases; experimentally determined. Predicted interactions:gene neighbourhood. Others: text-mining; co-expression; protein homology. **(B)** Ten interactive genes expression fold changes in EC tissue compared to NEC tissue. A positive LogFC indicates up-regulation.

To explore the potential targets of GhJMJs in cotton somatic embryogenesis, protein-protein interaction network was constructed for the homologous of GhJMJ24 and GhJMJ49 in *G. raimondii* (Figure 8). In *G. raimondii*, *GrJMJ24* is the ortholog of *GhJMJ24* and *GhJMJ49*. As shown in Figure 8A, GrJMJ24 was predicted to have complex interactions with ten proteins, including SUVH4(KYP) histone methyltransferases (Gorai.005G196900.1, Gorai.012G082600.1), SUVH6 (Gorai.008G231400.1, Gorai.004G192900.1), decreased and methylation 1 (DDM1, Gorai.008G068400.1), two cytosine-specific methyltransferase-related proteins CMT3 (Gorai.001G052000.1), CMT1 (Gorai.002G216500.1), one RNA-dependent RNA polymerase (RDR2, Gorai.008G296900.1), and two unknown proteins (Gorai.009G395600.1, Gorai.010G128800.1). KEGG enrichment analyses showed that the proteins in the putative network are mainly involved in the lysine degradation pathway (Supplementary Table S12). Furthermore, the expression patterns of the ten interactive genes were analyzed in EC and NEC tissues according to RNA-seq data. As shown in Figure 8B, all the genes were found to have positive logFC values, indicating the up-regulation of the gene expression in EC compared to NEC. In particular, CMT1, DDM1 and SUVH4 all displayed higher logFC values (logFC >1), suggesting their potential role in somatic embryogenesis (Supplementary Table S13). Altogether, these results provide further insights into the potential functions of the *GhJMJ* genes during somatic embryogenesis process in cotton.

## Discussion

As histone demethylases, *JmjC* gene members exist in the genomes of animals and plants, playing imperative roles in histone modifications and epigenetics (Klose et al., 2006; Chen et al., 2011). As the most important fiber crop, *G. hirsutum* has been and continue to be subjected to genetic engineering, mostly *via Agrobacterium*-mediated genetic transformation that is a process containing multiple integral steps of cotton tissue culture, and the dedifferentiation of NEC to EC is the most important step. Previous studies in rice and maize revealed that hypomethylation events were observed more frequently than hypermethylation in the process of tissue culture and somatic embryogenesis (Stroud et al., 2013; Stelpflug et al., 2014). A number of *G. hirsutum JmjC* genes have been found to respond to cold, salt, and osmotic stress to some extent (Zhang et al., 2020; Sun et al., 2021), the question of whether *JmjC* plays a role in cotton tissue culture and somatic embryogenesis remains unanswered until this study.

### The Cotton *JmjC* Gene Family Expanded but Remains Highly Conserved During Evolution

*G. hirsutum* is a naturally occurring allotetraploid plant, which was formed by spontaneous hybridization of two diploid cotton species that were related to *G. arboreum* (A2) and *G. raimondii* (D5) (Cedroni et al., 2003). In the present study, we identified 51 *GhJMJ* gene members in the *G. hirsutum* genome, which is less than anticipated considering homeologous *JmjC* genes present in the A and D subgenomes corresponding to *G. arboreum* (28 gene members) and *G. raimondii* (25 gene members), respectively, suggesting an event of gene loss that might have occurred in *G. hirsutum*, or an event of gene gain that might have occurred in either *G. arboreum* or *G. raimondii* since the formation of tetraploidization. In previous studies in *Arabidopsis* and rice (Lu et al., 2008), gene nomenclature and phylogenetic analysis of JmjC domain-containing proteins were carried out by following similar studies in humans. However, *JmjC* genes in plants have undergone sufficient divergence to develop plant-specific sub-families with specific functions (Huang et al., 2016). In this study, the *JmjC* gene members that were named according to the sequence of their chromosomal locations were divided into six subfamilies by the gene structure and the unrooted neighbor-joining (NJ) evolutionary tree. All the identified GhJMJ proteins share a JmjC domain that is known to be involved in the indispensable histone lysine demethylation. In all the six subfamilies, the Fe(II) and αKG binding sites within JmjC domain were highly conserved, and the specific structural feature in each group may underpin their distinct functions.

### Diverse Expression Patterns of *GhJMJ* Genes in *G. hirsutum*

Previous studies on plant *JmjC* genes have been focusing on their roles in growth, development and in response to environmental stresses (Klose et al., 2006; Kouzarides, 2007), but the question regarding whether *JmjC* could also be involved in the induction of somatic embryogenesis and plant regeneration remains unanswered. A number of genes that are differentially expressed during somatic embryogenesis in cotton have been documented, such as those that are involved in the production of auxins, indole-3-butyric acid (IBA), and those with the WUSCHEL-related homeobox (WUS) (Xiao et al., 2018; Sun et al., 2019; Fan et al., 2020; Wen et al., 2020). A recent report showed that a number of genes involving in the RdDM pathway of epigenetic, including MET1, CMT3, SUVH4, SUVH6, JMJ14, and DDM were up-regulated in the callus development process from NEC to EC in cotton (Li et al., 2019). In this study, for the first time, the *GhJMJ* genes were found to exhibit significant differential expression between NEC and EC tissues in cotton, and most of the *GhJMJ* genes were up-regulated when the tissue culture derived callus were in the transitionary phase from NEC to EC stage. In addition, two *GhJMJ* genes (*GhJMJ24* and *GhJMJ49*) in subfamily VI, were highly differentially expressed between EC and NEC tissues*.* Therefore, we can infer that GhJMJ genes, especially *GhJMJ24* and *GhJMJ49*, may perform important biological functions in cotton tissue culture by virtue of their highly elevated expression in EC tissue.

### Roles of *GhJMJ24* and *GhJMJ 49* During the Process of Somatic Embryogenesis

Plant regeneration via somatic embryogenesis involves many epigenetic changes, such as DNA methylation and histone modification (Klose et al., 2006; Kouzarides, 2007). It has been shown that the increase in CHH methylation in the transition process from NEC to EC is associated with the RdDM and the H3K9me2-dependent pathways, and the inhibition of DNA methylation in NEC could increase the number of somatic embryos regenerated in cotton tissue culture (Li et al., 2019). In *A. thaliana*, the repressive histone mark H3K9me2 is established mainly by the histone methyltransferase KRYPTONITE (KYP), and in turn KYP/SUVH4 and SUVH6 can catalyze H3K9me2, whereas CMT3 can catalyze non-CG methylation (including CHG and CHH) (Johnson et al., 2008; Enke et al., 2011). Meanwhile, SUVH binds to methylated DNA, and the chromodomain of CMT3 binds to H3K9me2, thus creating a self-reinforcing loop between the two epigenetic modifications (Johnson et al., 2008; Enke et al., 2011). In addition, the non-CG methylation could also be mediated by DDM1 and CMT2/3 involving the dimethylation of histone H3 at lysine 9 (H3K9me2) in heterochromatin regions (Du et al., 2012; Stroud et al., 2014). In addition, RNA-dependent RNA polymerase 2 (RDR2) was a necessary factor in 24-nt small interfering RNA (siRNA) biogenesis, which plays an indispensable role in the RdDM pathway (Fan et al., 2012). In *A. thaliana*, *AtIBM1/JMJ25* can negatively regulate the accumulation of H3K9 methylation, and the mutant of *AtIBM1/JMJ25* results in elevated levels of H3K9me2 and concomitant CHG hypermethylation in thousands of genic loci, giving rise to a variety of developmental phenotypes (Saze et al., 2008). However, both the *kyp/suvh4* and *cmt3* mutations suppress the detectable developmental phenotypes of *ibm1*/*jmj25* (Saze et al., 2008). In addition, *AtJMJ29* can directly target GLABRA 3 (GL3) and remove H3K9me2 on the GL3 locus, involving the trichome development *i*n *A. thaliana* (Hung et al., 2020). *AtIBM1/JMJ25*, *AtJMJ26*, *AtJMJ27,* and *AtJMJ29* have the conserved Fe(II) and αKG binding amino acids within the cofactor binding site and are active histone demethylases targeting H3K9me2 (Lu et al., 2008). Prior to our study, the roles of JmjC histone demethylases had not been investigated in cotton tissue culture process. In our study, the protein-protein interaction network of GhJMJ24 and GhJMJ49, included KYP/SUVH4, SUVH6, DDM1, CMT3, CMT1, and RDR2. With the use of nucleus markers and transient expression in *Arabidopsis* protoplasts, GhJMJ24 and GhJMJ49 were co-localized in the nucleus. As *GhJMJ24* and *GhJMJ4*9 are highly homologous to *AtIBM1/JMJ25* and *AtJMJ29*, their similar molecular mechanisms of epigenetic regulation are therefore conceivably assumed, which participate in controlling H3K9 methylation and DNA methylation and therefore alter gene expression in the nucleus during somatic embryogenesis in cotton.

### The Potential Regulatory Mechanisms of *GhJMJ* Genes Expression

The expression of gene can be regulated by multiple mechanisms, including transcriptional or posttranslational regulation. TFs temporarily and spatially regulate gene expression by binding to specific upstream sequence of a target gene, and hence control plant growth, development, and responses to environmental stimuli. In this study a number of important plant TFs, such as AP2/ERF, MYB, and NAC that potentially bind to *GhJMJs* and the putative TFBSs have been identified in cotton. NAC, C2H2 and Nin-like transcription factors might bind to the promoter of GhJMJ24 and GhJMJ49, and DNA binding with one finger (Dof) might bind to the promoter of GhJMJ49 distal from TSS. It is well recognized that AP2/ERF and MYB transcription factor families are the two largest TF families in plants, which play imperative regulatory roles in plant development, differentiation, and metabolism (Ambawat et al., 2013; Chu et al., 2017). Further, Dof proteins constitute a ubiquitous plant-specific TF family associated with diverse biological processes; the C2H2 zinc finger protein regulates plant responses to environmental stimuli, such as salinity, drought, and excessive light. The transcriptional regulations of *GhJMJs* by these well characterized TFs as outlined in this study represent the first step towards a good understanding on their role in inducting somatic embryogenesis, which needs further experimental verification.

MiRNAs promote translational repression or mRNA degradation through binding to the complementary sequences on their target mRNA transcript, involving in various aspects of plant growth and development (Gao et al., 2016). To our knowledge, there are scant work on *JmjC* gene expression and regulation mediated by miRNAs. In this study, all the presence of potential miRNA target sites were predicted in all *GhJMJs*, including numerous important miRNAs, such as miR156, miR157, and miR172, among which miR156 is a highly conserved plant miRNA and has been extensively studied because of its versatile roles in plant development (Yu et al., 2015; Yin et al., 2017). Previous studies have shown that miR156 involved in regulating somatic embryogenesis in citrus (Long et al., 2018). Further, miR156/SPLs and miR172/AP2 modules, which represent key regulatory hubs involved in plant phase transition, were modulated by SAP11 (Long et al., 2018; Rao et al., 2021). In wheat, embryogenic callus formation and somatic embryogenesis were found in association with a number of miRNAs, such as miR156, miR164, miR1432, miR398, and miR397 (Chu et al., 2016). In cotton, *GhmiR157a* negatively regulates *GhSPL10* and promotes callus induction by inducing ethylene and auxin responses, which further activates flavonoid biosynthesis to promote callus proliferation (Wang et al., 2018). In this study, how these predicted miRNAs are involved in *GhJMJ* gene expression and regulation during embryogenic callus formation in cotton requires further exploration.

However, the potential regulatory mechanisms of *GhJMJ* gene expression are yet to be elucidated in cotton. In this study, we analyzed the TFBSs and miRNA target sites which provide insights into the potential regulatory mechanisms of *GhJMJs* expressions in cotton.

## Conclusion

We systematically analyzed 51 predicted *GhJMJ* genes, including their gene structure, phylogeny, conserved domains and motifs, and expression profiles, with reference to their biological functions. *GhJMJ24* and *GhJMJ49* were predominantly expressed in EC compared to NEC in tissue culture, which might be regulated by transcription factors and miRNAs. Subcellular localization assays and interaction network analysis unraveled that GhJMJ24 and GhJMJ49 might play important roles in the transition from NEC to EC by reversing H3K9me2 methylation in the nucleus where they function by interacting with SUVH4, SUVH6, DDM1, CMT3, and CMT1. This study suggests that *GhJMJ* genes plays an important role in embryogenic callus formation in cotton, which warrants further investigation and a thorough understanding on their functional role would enable the development of efficient transformation systems in cotton and many other plant species.

## Data Availability

The original contributions presented in the study are included in the article/Supplementary Material, further inquiries can be directed to the corresponding authors.
