# Supplementary material for: Genome-wide Characterization of the JmjC Domain-Containing Histone Demethylase Gene Family Reveals GhJMJ24 and GhJMJ49 Involving in Somatic Embryogenesis Process in Cotton
Source: Front Mol Biosci. 2022 Apr 27;9:888983. doi: 10.3389/fmolb.2022.888983 (PMC9091307; doi:10.3389/fmolb.2022.888983)
Supplement: Supplementary file 2 [file DataSheet1.PDF]

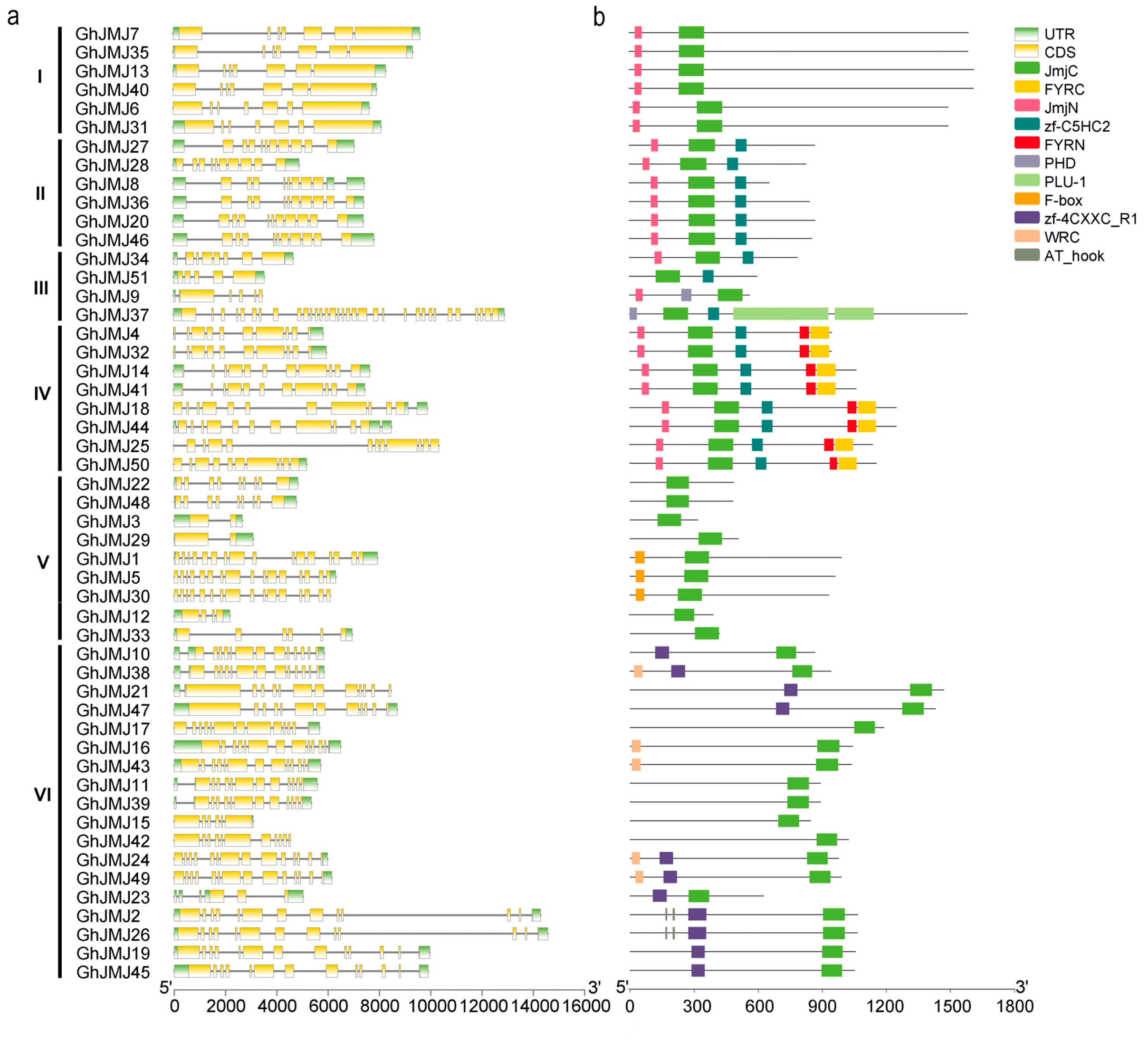

Supplementary Figure 1. Gene structure and conserved domains analysis of the GhJMJs. a Exon-intron structure of cotton GhJMJ genes. Exons and introns are shown by orange boxes and gray lines; the upstream and downstream regions are shown by pale green boxes. b Domain composition of cotton GhJMJ proteins. Domains are displayed in different colored boxes. The length of genes and proteins can be estimated using the scale at the bottom.

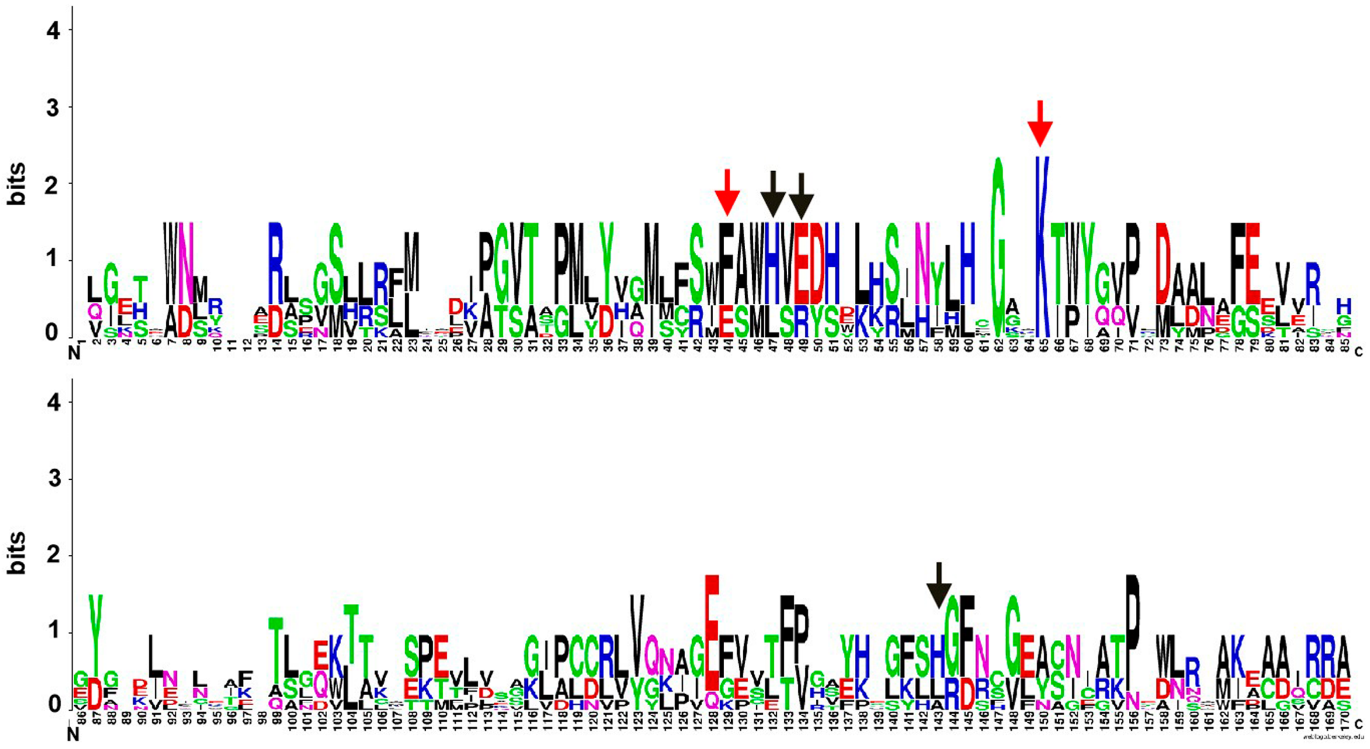

Supplementary Figure 2. The conservation of JmjC domains in the GhJMJ gene family. Fe(II) binding sites are shown with red arrowheads, and αKG binding sites are shown with black arrowheads.

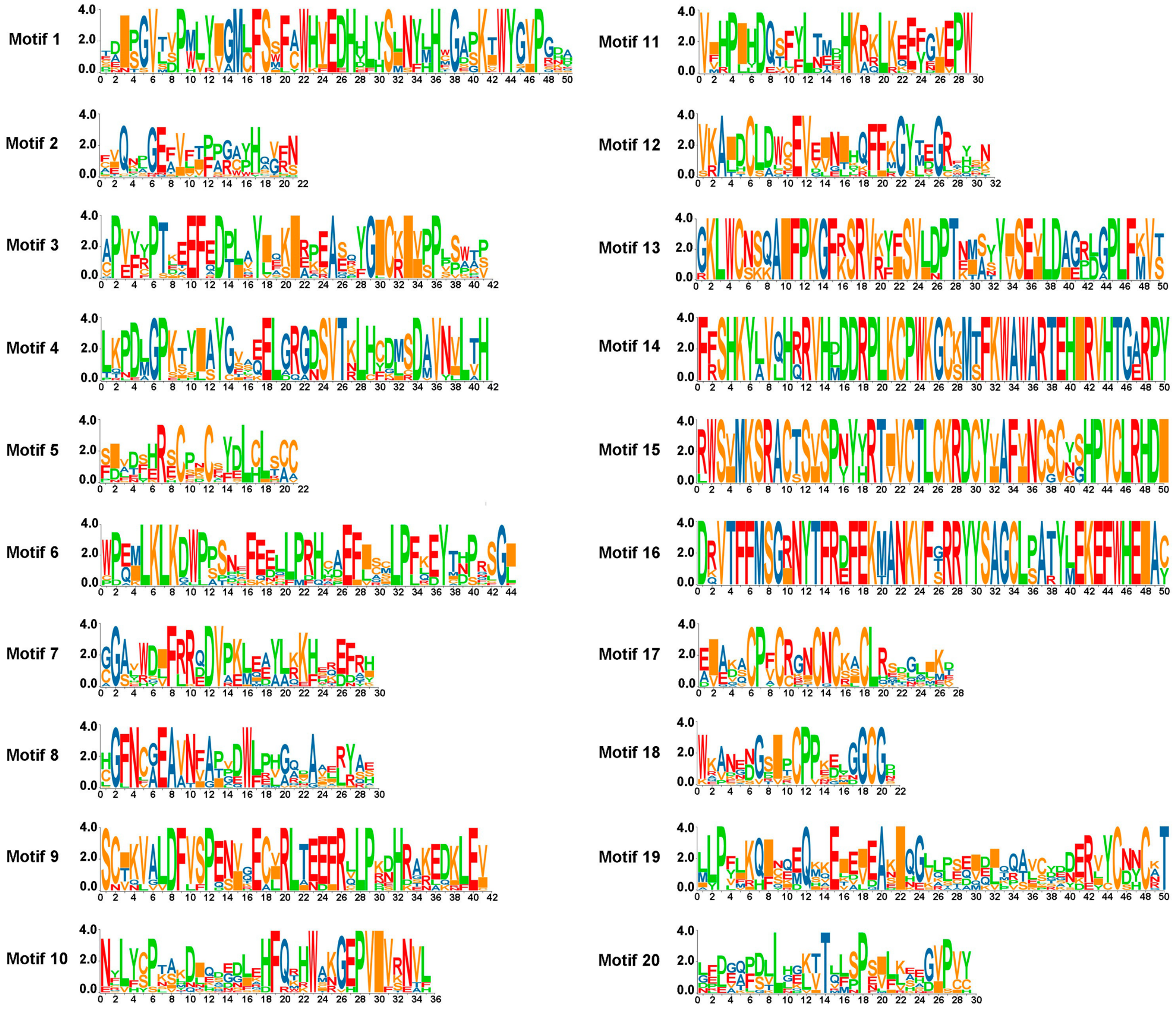

Supplementary Figure 3. Sequence logos of 20 motifs in the GhJMJ family generated by the application WebLogo.

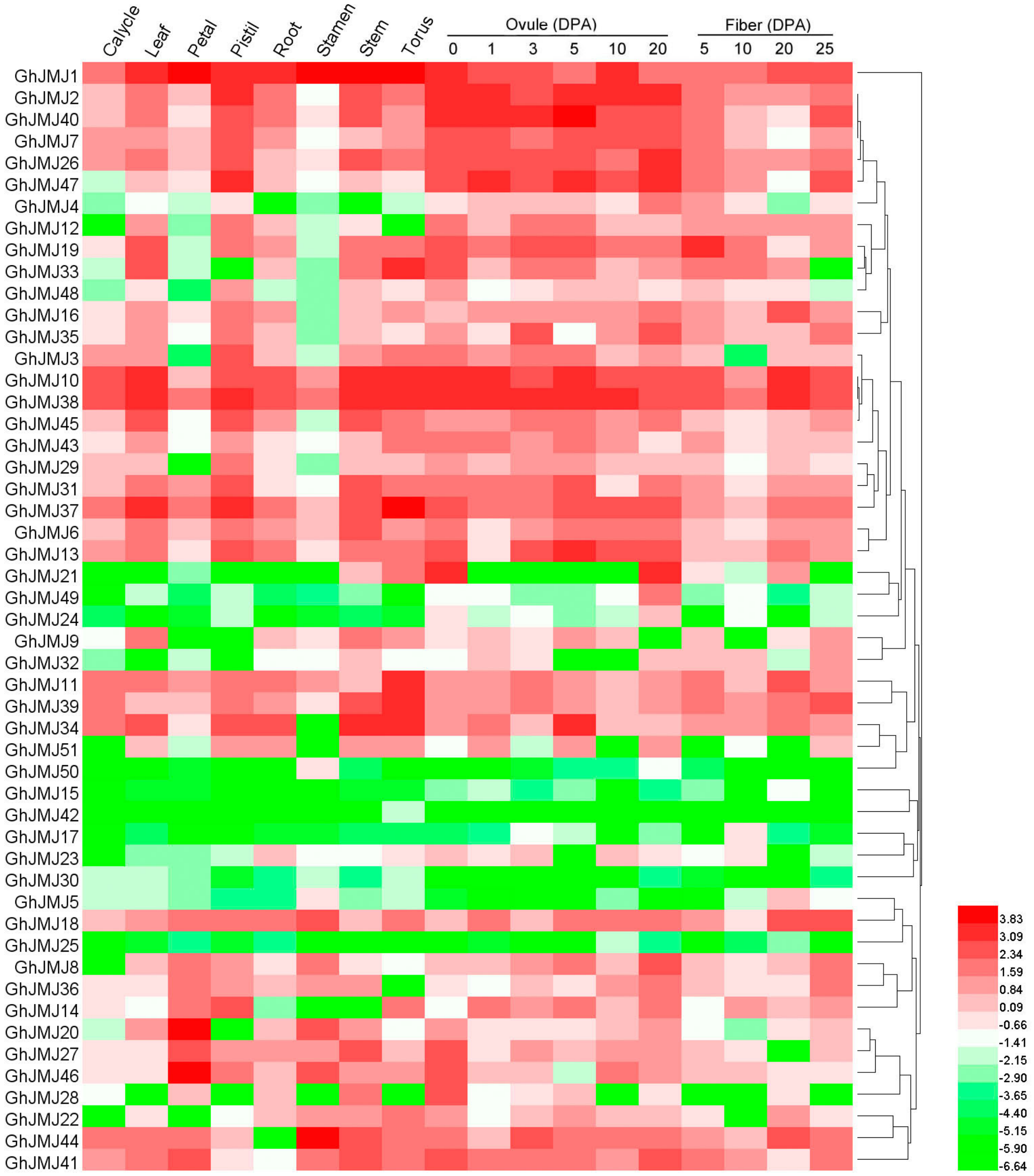

Supplementary Figure 4. Heat map of GhJMJ genes expression profiles in cotton different tissues.Scale bars represent the log2 transformations of the RPKM values. The red color represents high expression and green represents low expression. DPA is an acronym for days post-anthesis.

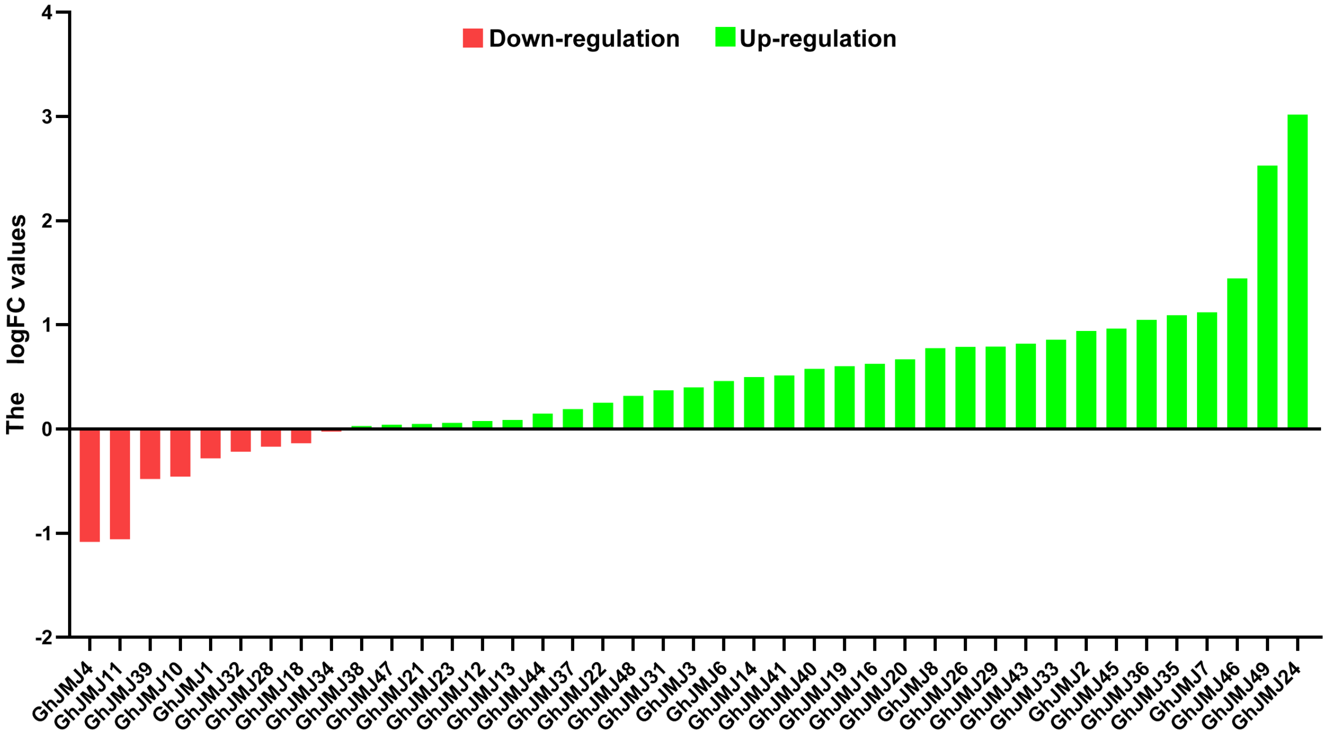

Supplementary Figure 5. 41 GhJMJs genes expression fold changes in EC/NEC. A positive LogFC indicates up-regulation; a negative LogFC indicates down-regulation.
